# Wee1 Inhibitor AZD1775 Combined with Cisplatin Potentiates Anticancer Activity against Gastric Cancer by Increasing DNA Damage and Cell Apoptosis

**DOI:** 10.1155/2018/5813292

**Published:** 2018-06-07

**Authors:** Dongshao Chen, Xiaoting Lin, Jing Gao, Lin Shen, Zhongwu Li, Bin Dong, Cheng Zhang, Xiaotian Zhang

**Affiliations:** ^1^Department of Gastrointestinal Oncology, Key laboratory of Carcinogenesis and Translational Research (Ministry of Education/Beijing), Peking University Cancer Hospital & Institute, Beijing 100142, China; ^2^Department of Pathology, Key Laboratory of Carcinogenesis and Translational Research (Ministry of Education/Beijing), Peking University Cancer Hospital & Institute, Beijing 100142, China

## Abstract

Based on the mechanisms by which Wee1 inhibitor and cisplatin played their own role, a promising strategy of Wee1 inhibitor combined with cisplatin was proposed, which was investigated in gastric cancer (GC). Either Wee1 inhibitor AZD1775 or cisplatin alone had a certain inhibitory effect on* in vitro* cell proliferation; however, the inhibitory effect was more significant when AZD1775 combined with cisplatin* in vitro* and* in vivo*. The underlying mechanisms unveiled that the increased DNA damage indicated by increased *γ*H2AX protein, as well as augmented cell apoptosis indicated by upregulated proapoptotic proteins, was responsible for the significant inhibitory effect of AZD1775 plus cisplatin. Moreover, compared to any single drug,* in vitro* cell migration and invasion abilities were further attenuated by AZD1775 combined with cisplatin. There were suggestive results that the potentiated cytotoxicity between AZD1775 and cisplatin deserved a deep exploration in the future.

## 1. Introduction

Gastric cancer (GC) is the second leading cause of cancer and cancer-related deaths in China [1]. Although conventional chemotherapy and emerging targeted therapy have brought survival benefits for patients, prognosis of GC remains poor due to the limited activity of monochemotherapy and insufficient choices of current clinically available treatment [2]. Thus, development of novel therapeutic strategies against GC, especially in the presence of chemotherapy, is in urgent need.

Wee1, a tyrosine kinase, serves as a crucial regulator of the G_2_/M checkpoint that keeps cells with DNA lesions from mitotic entry [3]. In the content of DNA damage, Wee1 phosphorylates and inactivates cyclin-dependent kinase 1 (CDK1) to safeguard the G_2_/M checkpoint [4]. Having been reported to be overexpressed and predicting poor prognosis in several cancer types (including GC) [5–8], Wee1 is considered to be a novel therapeutic target against GC.

Wee1 blockade, an emerging anticancer therapy among a range of cancer types [9–11], can abrogate the G_2_/M checkpoint and force cancer cells with unrepaired DNA lesions to enter into unscheduled mitosis and undergo DNA damage-mediated cell death, namely, mitotic catastrophe [4, 12]. In the light of mechanisms underlying Wee1 inhibition's anticancer actions, Wee1 blockade combined with DNA-damaging agents has been recently proposed in the treatment of cancer. AZD1775, a most common selective and potent Wee1 inhibitor [13], has been reported to synergize with various genotoxic drugs in the treatment of cancers [13–15]. However, therapeutic efficacy of Wee1 inhibitors combined with DNA-damaging agents against GC and its underlying mechanisms remain largely unknown. In this work, GC cell lines and xenografts were used to explore the therapeutic potential of a mainstream Wee1 inhibitor AZD1775 combined with cisplatin and its underpinning mechanisms. Our study sheds light upon the improvement of current therapy for GC and provides evidence for further clinical investigation.

## 2. Materials and Methods

We followed the methods of Chen et al. [2018] [16] in this section.

### 2.1. Reagents and Antibodies

AZD1775 and cisplatin were purchased from Selleck Chemicals (Houston, TX) and Hospira Australia Pty Ltd (Victoria, Australia), respectively. Reagents were formulated and stored following manufacturer's protocols for* in vitro* and* in vivo* experiments. Primary antibodies against cleaved caspase 3 (#9664), cleaved caspase 9 (#20750), cleaved PARP (#5625), *γ*H2AX (#9718), and secondary horseradish peroxidase- (HRP-) conjugated goat anti-rabbit (#7074) and anti-mouse antibodies (#7076) were purchased from Cell Signal Technology (CST, Danvers, MA). Antibodies against *β*-actin (#A5441) and Ki-67 (#ZM-0167) were purchased from Sigma-Aldrich (St. Louis, MO) and ZSGB-BIO (Beijing, China), respectively.

### 2.2. Cell Lines and Cell Culture

Human GC cell lines (MKN45, N87, and AGS) were kindly provided by Professor Youyong Lv (Peking University Cancer Hospital & Institute). Other cell lines (MGC803, SNU1, and KATOIII) were purchased from Shanghai Institutes for Biological Sciences (Shanghai, China) except for HGC27 (Bank of Chinese Academy of Sciences, Beijing, China) and SNU5 (American Type Culture Collection, Manassas, VA, USA). A majority of cell lines were cultured in RPMI 1640 medium (Gibco BRL, Gaithersburg, MD) except for SNU5 and N87, which were cultured in IMDM medium (Gibco BRL) and DMEM medium (Gibco BRL), respectively. All media were supplemented with 10% fetal bovine serum (FBS) (Gibco BRL) and 1% penicillin and streptomycin (HyClone, Logan, UT). Cells were incubated in a humidified incubator (37°C) with 5% CO_2_.

### 2.3. Cell Viability Assays

Cells (4,000 cells/well) were seeded into 96-well plates and allowed to adhere overnight in complete medium. Following drug treatment as indicated for 48 h, cell viability was measured using a Cell Counting Kit-8 (CCK-8) commercial kit (Dojindo laboratories, Tokyo, Japan) according to the manufacturer's protocol. Absorbance was measured at 450 nm by spectrophotometer. All experiments were repeated three times.

### 2.4. Cell Apoptosis Assay

Cells exposed to AZD1775 with/without cisplatin for 48 h were collected, washed in phosphate-buffered saline (PBS), and double-stained using Annexin V-Phycoerythrin (PE) and 7-amino-actinomycin (7-AAD) apoptosis detection kit (BD Biosciences, Erembodegem, Belgium) following the vendor's protocol. Samples were detected by flow cytometry within 1 h (BD Biosciences) and proportions of apoptotic cells were analyzed using the FlowJo version 7.6.1 software (FlowJo, Oregon).

### 2.5. Cell Cycle Assay

After exposure to AZD1775 with/without cisplatin for 24 h, cells were collected, washed with PBS, and fixed in 70% immediately prepared precooled ethanol overnight at 4°C. After washing with PBS three times, cells were stained with propidium iodide (PI)/RNase solutions using a commercial cell cycle detection kit (BD Biosciences) at room temperature for 15 min in the dark according to the instructions, followed by flow cytometry analysis within 1 h (BD Biosciences). Cell cycle distributions were assessed with ModFit version 3.0 software (Verity Software House, Topsham, ME).

### 2.6. Immunofluorescence Staining

Cells at a density of about 300,000 cells/ml were seeded on a 35 mm glass bottom dish (NEST, Jiangsu, China) and incubated overnight before treatment. After exposure to AZD1775 with/without cisplatin for 12 h, cells were fixed with 4% paraformaldehyde (Solarbio, Beijing, China) for 10 min, followed by permeabilization with 0.5% Triton X-100 (Amresco, Solon, OH) for 20 min and blocking for 30 min using 5% bull serum albumin (BSA) (Amresco). The primary antibody against *γ*H2AX (1:100) was added at 4°C overnight followed by incubation with Alexa Fluor 488-conjugated goat anti-rabbit immunoglobulin G (IgG) (Molecular Probes, Eugene, OR, 1:100) in the dark for 1 h and 4',6-Diamidino-2-Phenylindole (DAPI) (Beyotime, Jiangsu, China, 1:3,000) in the dark for 5 min. All reagents were diluted in PBS. Images were captured with the ZEN version 2012 software (Zeiss, Gottingen, Germany) using a laser scanning confocal microscope LSM 780 (Zeiss). The same exposure parameters were applied in all images. Three random fields were chosen to count *γ*H2AX-positive cells stained with more than 10 green foci as reported [17].

### 2.7. Cell Invasion and Migration Assays

Cells were pretreated with AZD1775 in the presence or absence of cisplatin for 24 h. 20,000-30,000 cells in 100 *μ*l of serum-free medium were added to the upper chamber with/without precoated Matrigel (Corning, New York, NY). Medium supplemented with 10% FBS was added to the lower chambers. After incubation for 48 h and 24 h for invasion/migration assays, respectively, the invaded and migrated cells in the lower chambers were fixed and stained with crystal violet and counted under a microscope.

### 2.8. Immunoblotting Analysis

After drug treatment, GC cells and tumor tissues were lysed using a CytoBuster protein extraction reagent (Merck Millipore, Darmstadt, Germany) in the presence of protease and phosphatase inhibitor cocktail tablets (Roche, Basel, Switzerland). Protein concentration was measured with a bicinchoninic acid (BCA) protein assay kit (Beyotime). Soluble lysates were subjected to sodium dodecyl sulfate- (SDS-) polyacrylamide gel electrophoresis (PAGE) and transferred to polyvinylidene fluoride (PVDF) membranes (Merck Millipore). After blocking with 5% BSA (Amresco), membranes were probed with primary antibodies (1:1,000 diluted in blocking solutions except 1:10,000 for *β*-actin) at 4°C overnight followed by secondary antibodies (1:2,000) at room temperature for 1 h. Signals were visualized using Amersham Imager 600 (GE Healthcare, Chicago, IL) after incubation with Clarity Western ECL substrate (Bio-Rad, Hercules, CA).

### 2.9. *In Vivo* Studies

MGC803 cells were detached with trypsin (Gibco BRL) and resuspended with PBS to a final concentration of 2×10^7^ cells/ml. Then, 100 *μ*l cell suspension was inoculated subcutaneously in the right flank of 6-week-old female nonobese diabetic/severe combined immunodeficiency (NOD/SCID) mice (Vital River Laboratories, Beijing, China). When tumor volume reached approximately 150-250 mm^3^, mice bearing MGC803 cells were randomly assigned to treatment groups (*n* = 5) and given PBS (100 *μ*l, daily, by oral gavage) or AZD1775 (30 mg/kg/d, daily, by oral gavage) alone or the combination of AZD1775 and cisplatin (3 mg/kg, twice a week, i.p.) for 21 days. Tumor size and body weight were measured every three days and tumor volume (V) was calculated by the following formula: V = L × W^2^/2 (L, long diameter of the tumor; W, short diameter of the tumor). After the final drug administration, mice were sacrificed and tumors were stripped for successive assays. All animal experiments were approved by Peking University Cancer Hospital's Institutional Animal Care and Use Committee and complied with the internationally recognized Animal Research: Reporting of* in vivo* Experiments guideline.

### 2.10. Immunohistochemistry (IHC)

After dewaxing, hydration, endogenous peroxidase removal, antigen retrieval (EDTA buffer pH9.0, high pressure and high temperature using a pressure cooker for 10 min), and blocking with 5% BSA, 4 *μ*m thick formalin-fixed and paraffin-embedded (FFPE) sections were incubated with the primary anti-Ki-67 antibody (1:300) at 4°C overnight followed by IgG/HRP polymer (ZSGB-BIO) and diaminobenzidine substrate (Gene Tech, Shanghai, China) complying to protocols. Two pathologists from the Department of Pathology in Peking University Cancer Hospital & Institute independently evaluated staining results as described in our previous study [18].

### 2.11. Statistical Analysis

All data were representative of 3 independent experiments and expressed as means ± SD. Differences between groups were analyzed by one-way or repeated-measures ANOVA using SPSS version 20.0 software (SPSS Inc., IL, USA) and *P* < 0.05 was considered statistically significant.

## 3. Results

### 3.1. Wee1 Inhibitor AZD1775 Combined with Cisplatin Further Inhibited Growth in GC Cells

**Figure 1:**
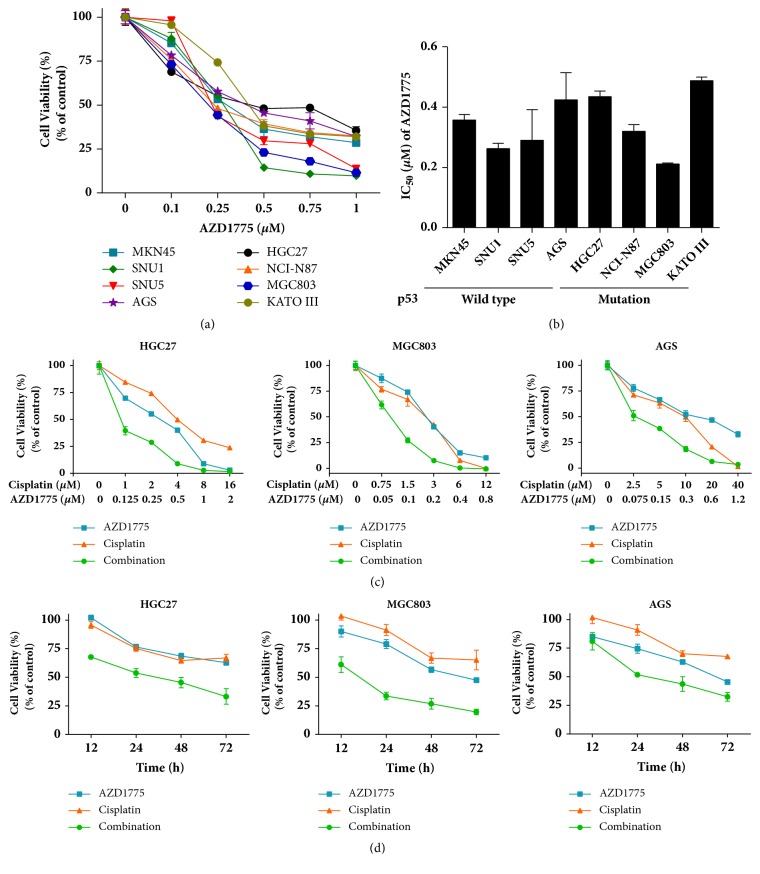
**Wee1 inhibitor AZD1775 combined with cisplatin further inhibited growth in GC cells**. (a) GC cells were treated with AZD1775 in a dose-dependent manner for 48 h. (b) IC_50_ values of AZD1775 among GC cell lines with p53 mutation and p53 wild type. Error bar, 95% confidence interval. (c) HGC27, MGC803, and AGS cells were treated with AZD1775 in the presence or absence of cisplatin as indicated for 48 h. (d) GC cells were exposed to AZD1775 (0.2 *μ*M) with/without cisplatin (2 *μ*M). Cell viability was determined by CCK-8 assay. Data are expressed as mean ± SD and are representative of three independent experiments.

To determine the therapeutic potential of Wee1 inhibitor-cisplatin combination against GC* in vitro*, a series of GC cell lines as indicated were treated with a widely used Wee1 inhibitor AZD1775 in the absence or presence of cisplatin. The p53 status is a controversial biomarker to predict sensitivity of cancer cells to Wee1 inhibitor [19–21]. Our data demonstrate that AZD1775 alone was cytotoxic across a broad panel of GC cell lines in a p53-independent manner, with IC_50_ values similarly ranging from 0.2 to 0.5 *μ*M in GC cells harboring either p53 mutation or wild type (Figures 1(a) and 1(b)). Of interest, AZD1775 at a clinically achievable concentration [22] combined with cisplatin yielded higher antiproliferative efficiency compared to their monotherapies (Figures 1(c) and 1(d)), indicating an augmented cytotoxicity of AZD1775 in the combination with cisplatin against GC cells.

### 3.2. AZD1775 Potentiated Cisplatin's Cytotoxicity through DNA Damage, Apoptosis, and G_2_/M Checkpoint Inactivation in GC Cells

**Figure 2:**
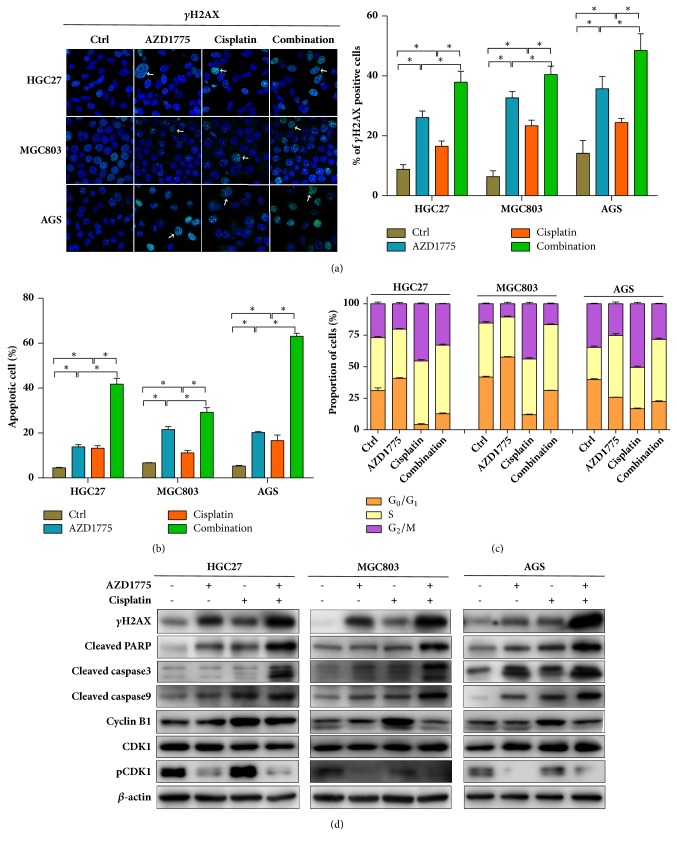
**AZD1775 potentiated cisplatin's cytotoxicity through DNA damage, apoptosis, and G**_2_ **/M checkpoint inactivation in GC cells**. HGC27, MGC803, and AGS cells were exposed to AZD1775 (0.2 *μ*M) with/without cisplatin (2 *μ*M). (a) *γ*H2AX-positive cells were counted with confocal immunofluorescence assays (green for *γ*H2AX; blue for DAPI-stained nuclei; scale bars, 20 *μ*m). ((b) and (c)) Percentages of apoptotic cells and cell cycle distributions were determined by flow cytometry following staining with Annexin V/7-AAD and PI/RNase buffers, respectively. (d) Expressions of indicated proteins were measured using Western blot. Data are expressed as mean ± SD and are representative of three independent experiments. *∗* indicates *P* < 0.05 by ANOVA analysis.

Induction of DNA damage has been reported as a primary cytotoxic consequence of Wee1 inhibitor in cancers [11]. To investigate mechanisms underlying augmented cytotoxicity of AZD1775 and cisplatin in GC, impacts of AZD1775 with/without cisplatin, a typical DNA-alkylating agent, on DNA damage were assessed. Our data reveal that the foci formation and protein level of *γ*H2AX, a representative marker of DNA double-strand breaks [23], were increased by administration of AZD1775 and these effects were potentiated in the presence of cisplatin (Figures 2(a) and 2(d)), suggesting that augmented DNA damage might be responsible for strengthened effects of AZD1775 plus cisplatin on GC growth inhibition.

AZD1775 or cisplatin's anticancer activity relies on the induction of apoptosis following DNA damage responses [8, 24–26]. Thus, we assayed the apoptotic changes after exposure to AZD1775 with/without cisplatin. Monotherapy of AZD1775 or cisplatin induced apoptosis in GC cells, while more apoptosis was induced in their combination group than single-agent groups (Figures 2(b) and S2). Molecular investigations reveal a more prominent upregulation of cleaved PARP, caspase 3, and caspase 9 in GC cells subjected to AZD1775-cisplatin combination compared to their monotherapy (Figure 2(d)). These data unveil the presence of enhanced apoptosis by coadministration of AZD1775 and cisplatin in GC cells. Taken together, a better response of GC cells to AZD1775 in the combination with cisplatin might be, at least partially, due to the increased DNA damage and subsequent apoptosis induction.

Since Wee1 inhibitors function on G_2_/M checkpoint [4, 27, 28], cell cycle alterations were evaluated in cells treated with AZD1775 with/without cisplatin. Our findings uncover cisplatin-induced G_2_/M cell cycle arrest indicated by increased cells arrested at G_2_/M phase and cyclin B1 (Figures 2(c), 2(d), and S2). Intriguingly, AZD1775 plus cisplatin promoted cell progression through G_2_/M phase compared to cisplatin monotherapy marked by reduced cell populations at G_2_/M phase, pCDK1, and cyclin B1 (Figures 2(c), 2(d), and S2). These data suggest that AZD1775-inactivated G_2_/M checkpoint also contributed to augmented anticancer effects of AZD1775-cisplatin combination in GC cells.

### 3.3. AZD1775 Combined with Cisplatin Further Attenuated Invasion and Migration Abilities in GC Cells

**Figure 3:**
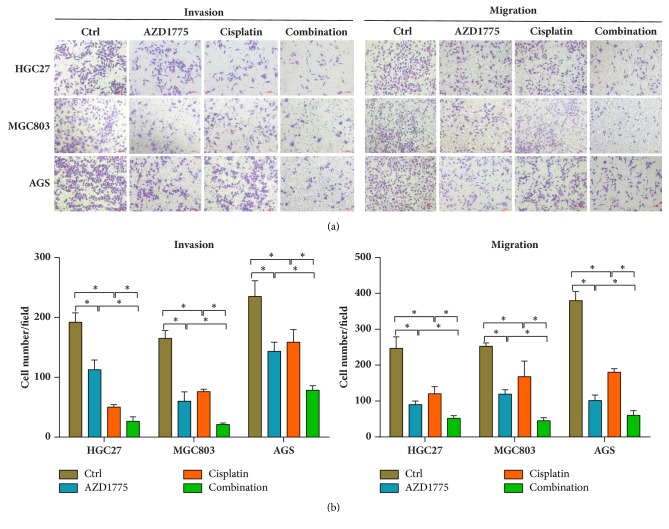
**AZD1775 combined with cisplatin further attenuated invasion and migration abilities in GC cells**. ((a) and (b)) After treatment with AZD1775 (0.2 *μ*M) in the presence or absence of cisplatin (2 *μ*M) in HGC27, MGC803, and AGS cells for 24 h, capacity of invasion and migration was detected using Transwell assays with/without Matrigel. Scale bars, 100 *μ*m. Data are expressed as mean ± SD and are representative of three independent experiments. *∗* indicates *P* < 0.05 by ANOVA analysis.

Apart from cellular growth inhibition, Wee1 blockade has also been reported to suppress cancer progression, and high expression of Wee1 is identified as a predictor of poor long-term prognosis that often results from metastasis [7, 29]. Thus, invasion and migration abilities were compared among GC cells exposed to AZD1775 with/without cisplatin. Figures 3(a) and 3(b) show decreased invasion and migration abilities after treatment with AZD1775 or cisplatin alone, while a more prominent effect was seen in AZD1775-cisplatin combination, indicating that AZD1775 combined with cisplatin exerted stronger capability in reducing invasion and migration of GC cells.

### 3.4. AZD1775 Combined with Cisplatin Further Reduced GC Tumor Growth* In Vivo*

**Figure 4:**
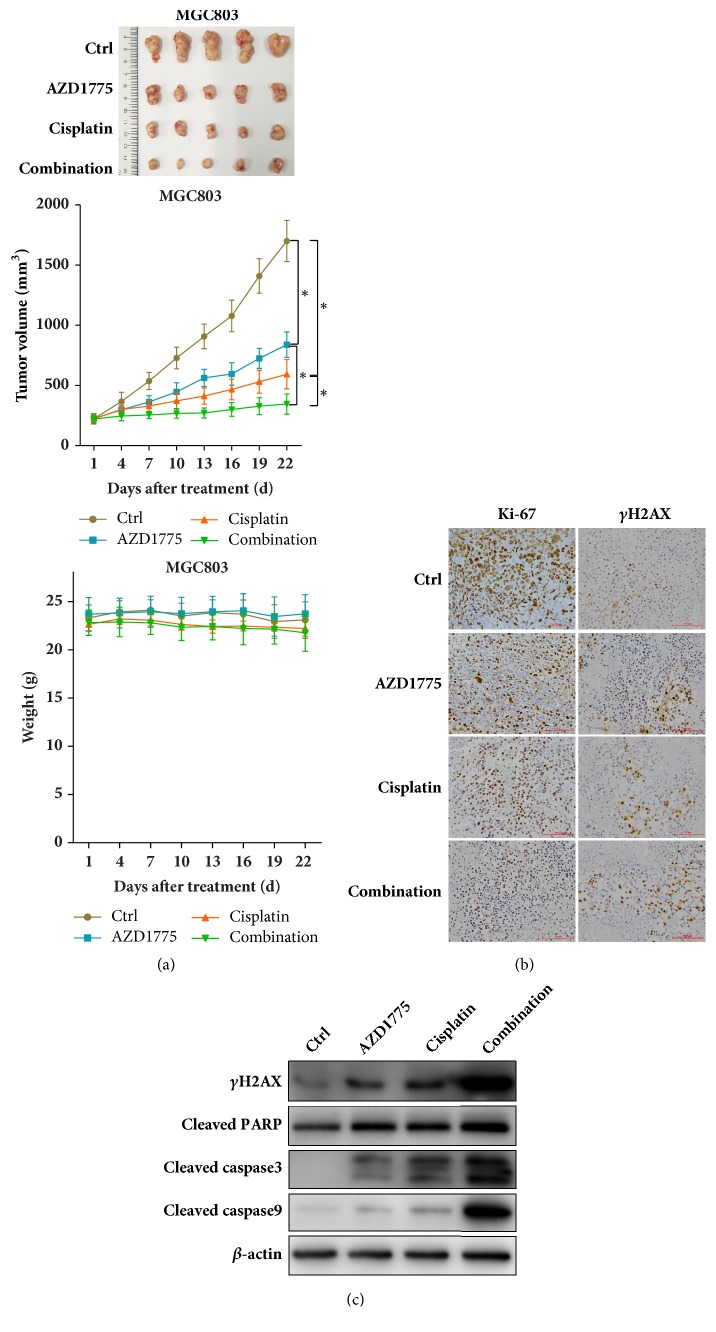
***AZD1775 combined with cisplatin further reduced GC tumor growth in vivo***. (a) AZD1775 (30 mg/kg/d, by oral gavage) with/without cisplatin (3 mg/kg, twice weekly, i.p.) was given to mice bearing MGC803 tumors for 21 days (*n* = 5 per group). Tumor volume and mice weight were measured every three days after treatment and xenograft growth curves were shown. Data are expressed as mean ± SD. *∗* indicates *P* < 0.05 by repeated-measures ANOVA. (b) FFPE sections stained with Ki-67 and *γ*H2AX for IHC analysis. Original magnification, 200x. (c) Tumor lysates were immunoblotted for indicated proteins.

Based on potentiated anticancer effects of AZD1775 plus cisplatin observed in GC cell lines,* in vivo* experiments were performed in mice xenografts harboring MGC803 cells to determine the therapeutic potentials of this combination strategy. AZD1775 or cisplatin alone repressed GC tumor growth to some extent, while their coadministration exerted a greater antigrowth efficiency than their single-agent groups without weight loss (Figure 4(a)), which was further validated by the lowest proliferation rate marked by Ki-67 immunostaining in GC tumors cotreated with AZD1775 and cisplatin (Figure 4(b)). In parallel to the* in vitro* findings, DNA damage (indicated by upregulated foci formation and protein levels of *γ*H2AX; Figures 4(b) and 4(c)) and apoptosis (marked by increased cleaved PARP, caspase 3, and caspase 9; Figure 4(c)) were induced by AZD1775 or cisplatin alone, while they were further increased in AZD1775-cisplatin combination. Therefore, Wee1 inhibitor combined with cisplatin achieved an enhanced therapeutic efficacy with good safety, at least partially, through increased DNA damage and apoptosis in GC tumors.

## 4. Discussion

Due to its high heterogeneity, current gastric cancer prevention and management are accompanied with serious difficulties, including limited chemotherapeutic responses, few targeted drugs, and poor prognosis. Hence, novel therapeutic options, especially combined with conventional chemotherapy, are of urgent demand to be developed for GC treatment. Intriguingly, Wee1 blockade, especially in combination with genotoxic chemotherapies, is emerging as a new therapeutic strategy and has been subjected to various clinical trial investigations among a variety of cancers [22]. Monotherapy of Wee1 inhibitor has been reported to repress growth, invasion, and migration in GC preclinical models [8]. However, the anti-gastric cancer potentials as well as the underlying mechanisms of targeting Wee1 with DNA-damaging agents, particularly cisplatin, remain largely unknown.

In GC cell lines and xenografts, we for the first time demonstrated the potentiated cytotoxicity of AZD1775, a widely used Wee1 inhibitor, in the presence of cisplatin, which might be due to an increased DNA damage and subsequent apoptotic cell death (Figures 1, 2, and 4). As reported, Wee1 inhibition results in dephosphorylation and activation of CDK1 followed by impaired G_2_/M checkpoint, premature mitosis, and DNA damage-associated cell death [4]. Likewise, AZD1775 inactivated G_2_/M checkpoint to abrogate G_2_/M arrest induced by cisplatin (Figures 2(c), 2(d), and S2) [28], indicating DNA damage enhancement of AZD1775-cisplatin combination partially due to G_2_/M checkpoint abrogation in our work. Beyond replication stress initiated by G_2_/M checkpoint abrogation, AZD1775-induced DNA damage has also been attributed to AZD1775's effect on dephosphorylation and activation of CDK2 during S phase which regulates overall timing of DNA replication [11, 30]. AZD1775 can cause deficiency in homologous recombination repair [31], which serves as another approach to AZD1775-induced DNA damage. These actions by AZD1775 are all optimal in the context of excessive DNA lesions [32, 33], which provides rationales for AZD1775 combined with DNA-damaging agents. However, what is responsible for enhanced DNA damage against GC observed in our study remains to be deciphered. On the other hand, DNA damage often leads to cell death through apoptosis induction [34, 35]. AZD1775-administrated strategies have been reported to yield cellular lethality through DNA damage and following apoptosis in a plenty of cancers [8, 17, 24]. Accordingly, our molecular investigations (Figures 2(d), 4(b), and 4(c)) unveil a consistent upregulation of cleaved PARP, caspase 3, and caspase 9, which has been observed in cancers treated with AZD1775 or cisplatin [8, 10, 17, 21]. Of note, *γ*H2AX frequently used as a DNA damage marker can also increase in the context of later apoptosis [36–38]; thus, other DNA damage experiments such as comet assay [39] warrant reliance of AZD1775-cisplatin combination's cytotoxicity on DNA damage. Changes in *γ*H2AX assessed with a system lacking apoptotic proteins like cleaved caspase 3 might also work [36].

Seeking potential predictive biomarkers is critical for optimizing therapeutic efficacy of AZD1775 combined with cisplatin. Of interest, p53 mutation is one of best-studied predictive biomarkers for Wee1 inhibition, yet whether cancers harboring p53 mutation have a better response to Wee1 inhibition-based strategies remains controversial [40]. Impaired p53 expressions or functional loss in cancer has been reported to induce deficiency in G_1_/S checkpoint, which may confer more reliance on a functional G_2_/M checkpoint for DNA repair [4]. Since AZD1775 exerted its anticancer cytotoxicity partially by inactivating G_2_/M checkpoints, the efficacy of AZD1775-based treatment was higher in p53-mutated than p53-wild type tumors [20, 21, 41, 42]. Nevertheless, disputable opinions exist demonstrating that p53 status is indispensable for AZD1775's anticancer activity [10, 19, 24]. DNA damage rather than premature mitosis (a typical phenotype of G_2_/M checkpoint defects) has recently been proven to be the primary cytotoxic consequence of AZD1775 in some cases [11, 30]. As mentioned above, apart from through a p53-reliant G_2_/M checkpoint defect, AZD1775 can also generate DNA damage-associated cytotoxicity through a p53-independent manner, such as homologous recombination defect and DNA replication disruption by inactivating CDK2 [11, 30, 31]. Consistent with these studies, GC cells with p53 mutation and wild type have a similar response to Wee1 blockade [8] and our findings also reveal that the response to AZD1775 and enhanced efficacy of AZD1775 plus cisplatin were independent of p53 status (Figure 1). Moreover, we observe that Wee1 expression failed to predict sensitivity of AZD1775 with/without cisplatin (Figure S1), which remains in dispute in different researches [24, 43]. Due to high heterogeneity in GC, the correlation of p53 status and Wee1 expression in GC's response to Wee1 inhibitors-contained therapy deserves to be further studied in expanded GC models, such as patient-derived xenografts. Predictive value of alternative newfound targets of AZD1775, such as PLK1 [44], is also worthy of an investigation.

Apart from growth, Wee1 plays a critical part in cancer progression. Wee1 overexpression has been reported to protect endothelial cells of colorectal cancer from liver metastases and suppress invasion and migration in GC cells [8, 45]. Cisplatin-mediated metastasis suppression is also observed in breast cancer [46] and ovarian cancer [47]. However, impacts of Wee1 inhibitor combined with cisplatin on metastasis remain unclear. Our data demonstrate reduced invasion and migration abilities in GC cells treated with AZD1775 or cisplatin, especially in their combination (Figure 3). However, molecular mechanisms underlying this combination-reduced invasion and migration abilities remain to be explored.

In conclusion, the Wee1 inhibitor AZD1775 combined with cisplatin potentiated cytotoxicity through increased DNA damage and subsequent apoptotic cell death in GC cell lines and xenografts. AZD1775 and cisplatin both attenuated the invasion and migration abilities in GC cells, while their combination exerted augmented effects. Our data provide evidence for therapeutic potentials of Wee1 inhibitors plus cisplatin, and this promising combination strategy is expected to be investigated in the clinic.

## Data Availability

The cell viability, cell apoptosis, cell cycle, immunofluorescence, Western blot, Transwell assay, immunohistochemistry, and animal data used to support the findings of this study are included within the article and supplementary information file.

## Supplementary Materials

Supplementary Materials Figure S1: Wee1 expression level among GC cell lines. Figure S2: impacts of AZD1775 combined with cisplatin on apoptosis and cell cycle in GC cells. ((a) and (b)) After drug treatment, apoptotic and cell cycle analyses were determined by flow cytometry in GC cells stained with Annexin V-PE/7-AAD and PI/RNase buffers, respectively.
