# Supplementary figures and images for: Wee1 Inhibitor AZD1775 Combined with Cisplatin Potentiates Anticancer Activity against Gastric Cancer by Increasing DNA Damage and Cell Apoptosis
Source: Biomed Res Int. 2018 Jun 7;2018:5813292. doi: 10.1155/2018/5813292 (PMC6011131; doi:10.1155/2018/5813292)

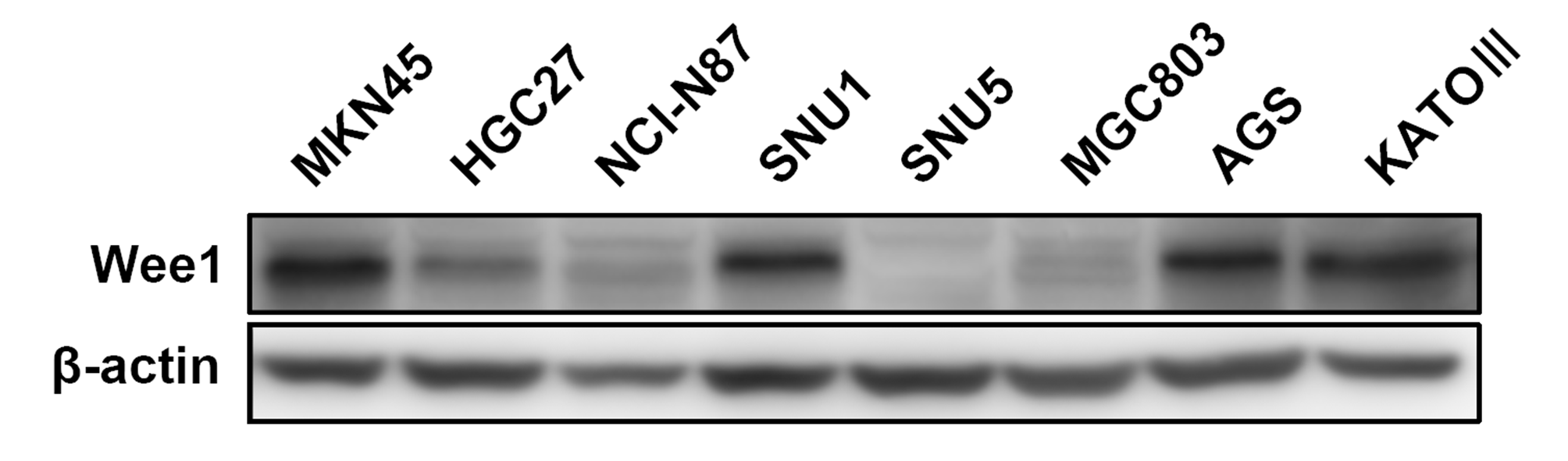

Supplement: Supplementary Materials — Figure S1: Wee1 expression level among GC cell lines. Figure S2: impacts of AZD1775 combined with cisplatin on apoptosis and cell cycle in GC cells. ((a) and (b)) After drug treatment, apoptotic and cell cycle analyses were determined by flow cytometry in GC cells stained with Annexin V-PE/7-AAD and PI/RNase buffers, respectively. [file 5813292.f1.zip › figure S1_BMRI_2310326.tif]

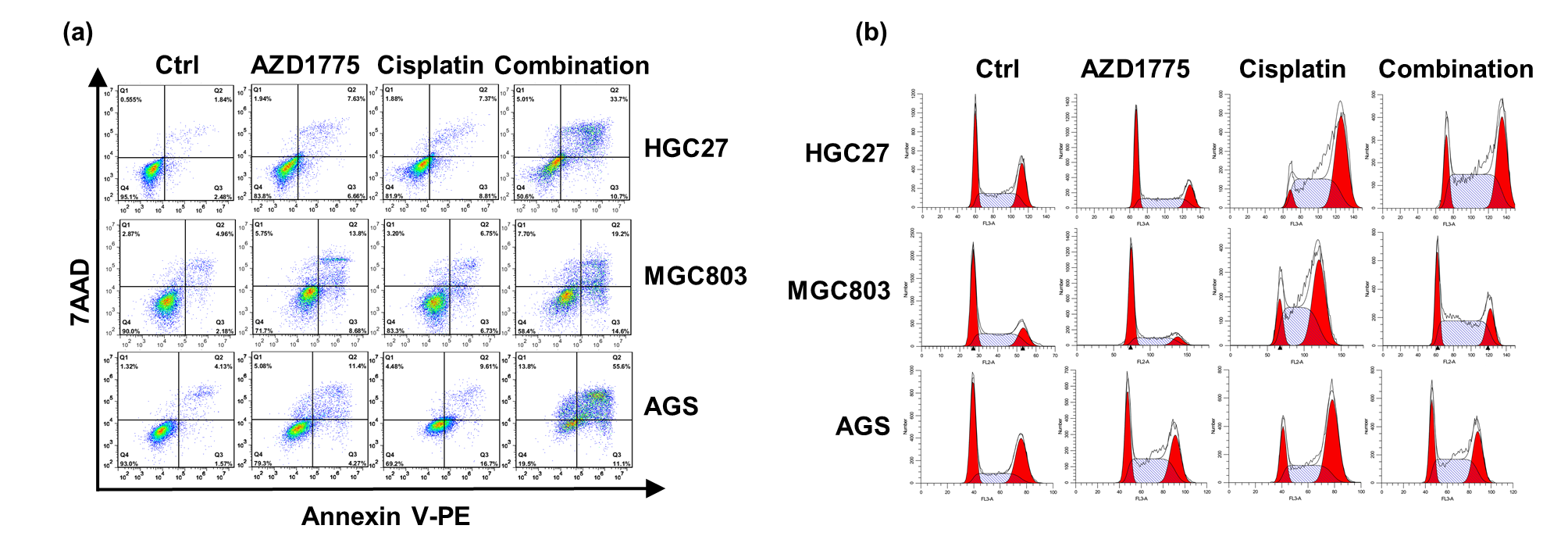

Supplement: Supplementary Materials — Figure S1: Wee1 expression level among GC cell lines. Figure S2: impacts of AZD1775 combined with cisplatin on apoptosis and cell cycle in GC cells. ((a) and (b)) After drug treatment, apoptotic and cell cycle analyses were determined by flow cytometry in GC cells stained with Annexin V-PE/7-AAD and PI/RNase buffers, respectively. [file 5813292.f1.zip › figure S2_BMRI_2310327.tif]
